# *Acinetobacter baumannii*: Virulence Strategies and Host Defense Mechanisms

**DOI:** 10.1089/dna.2021.0588

**Published:** 2022-01-12

**Authors:** Varnesh Tiku

**Affiliations:** Vir Biotechnology, San Francisco, California, USA.

**Keywords:** *Acinetobacter baumannii*, virulence mechanisms, bacterial infection, host immune responses, inflammation

## Abstract

*Acinetobacter baumannii* is a highly antibiotic-resistant bacterial pathogen known to cause severe life-threatening infections, including pneumonia, meningitis, and sepsis. Recent emergence of this bacterium as a serious nosocomial pathogen has led to categorization of *A. baumannii* as a “high-priority” pathogen by the World Health Organization (WHO), for which research efforts are urgently required to develop therapeutic interventions. Some of the properties that make *A. baumannii* a serious pathogen include its capacity to tolerate high levels of stress and enhanced expression of efflux pumps that enable high degrees of antibiotic resistance. Virulence mechanisms employed by *A. baumannii* to establish successful infection and host responses elicited against *A. baumannii* to counter the infection are discussed in detail in this article.

## Introduction

*A**cinetobacter baumannii* is a Gram-negative opportunistic pathogen that has gained enormous attention in the recent years. *A. baumannii* belongs to the genus *Acinetobacter*, which comprises more than 50 species, majority of which are nonpathogenic (Al Atrouni *et al.*, 2016). The most common species that have been linked with infections are *A. baumannii*, *Acinetobacter calcoaceticus*, and *Acinetobacter lwoffii* (Dijkshoorn and van der Toorn, 1992). Extensive clinical and animal model studies have clearly established *A. baumannii* as the most pathogenic bacteria in the genus *Acinetobacter* (Chusri *et al.*, 2014; Wong *et al.*, 2017). It has become one of the leading causes of nosocomial infections worldwide, including hospital-acquired pneumonia, and skin and urinary tract infections. *A. baumannii* is highly resistant to multiple antibiotics leading to its classification by the World Health Organization (WHO) as priority one threat to human health worldwide (Tacconelli and Margrini N, 2013). The pathogen employs various strategies to curb the effects of antibiotics, including expression of β-lactamases, multidrug efflux pumps, and aminoglycoside-modifying enzymes (Gordon and Wareham, 2010).

Another major attribute leading to widespread persistence of *A. baumannii* in nosocomial environments is its ability to resist severe conditions that are detrimental for other pathogens, thus conferring *A. baumannii* with a survival advantage. It can endure disinfection (Hassan *et al.*, 2013) and extended periods of desiccation (Antunes *et al.*, 2011). Moreover, *A. baumannii* is known to form robust biofilms both within the host (Thompson *et al.*, 2014) and also on abiotic surfaces such as hospital devices, which can further contribute to infection (Greene *et al.*, 2016b). Bacterial colonization in these biofilms increases extracellular stress tolerance and thus enhances bacterial persistence (Greene *et al.*, 2016a). Association of *A. baumannii* with hospital devices and equipment has become a major contributor to infection and has been linked to ventilator-associated pneumonia, catheter-associated urinary infections etc. (Wong *et al.*, 2017). Over the last few years many studies have been directed toward understanding the pathobiology of *A. baumannii.* A number of virulence mechanisms and host responses to infection have been uncovered, thus advancing our understanding of *A. baumannii* infection as detailed in the following sections.

## Major Virulence Strategies Employed by *A. baumannii*

**FIG. 1.**
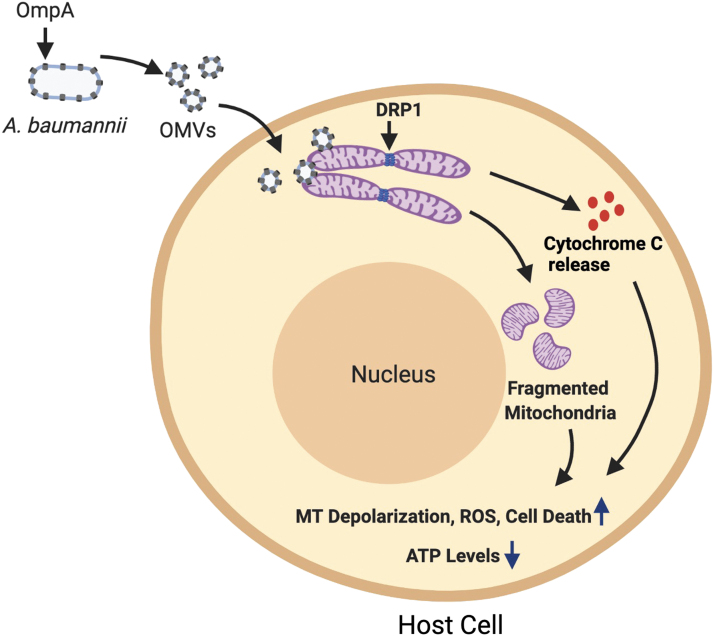
*Acinetobacter baumannii* secretes OMVs containing OmpA that induce mitochondrial damage. Cytotoxic OMVs containing OmpA are secreted by *A. baumannii*, which deliver OmpA to mitochondria. OmpA induces fragmentation of the mitochondrial network driven by the host protein DRP1, which is the master regulator of mitochondrial fission. OmpA-mediated mitochondrial fragmentation is accompanied with enhanced ROS levels, decreased ATP levels, and increased mitochondrial depolarization. OmpA-mediated damage to host cell mitochondria also leads to the release of the mitochondrial protein cytochrome C. Release of cytochrome C induces apoptotic cell death in host cells. Figure created with BioRender.com. OMVs, outer membrane vesicles; ROS, reactive oxygen species.

Multiple virulence factors and mechanisms have been described that *A. baumannii* employs to induce host damage. One of the major classes of proteins that has evolved from several studies has been porins. Porins are outer membrane proteins that modulate cellular permeability. OmpA is a major porin protein present in the outer membrane of *A. baumannii*. It serves multiple functions, including adhesion to host epithelial cells, resistance against complement killing, and biofilm formation (Schweppe *et al.*, 2015). OmpA protein is released through outer membrane vesicles (OMVs) of *A. baumannii* and targets mitochondria in host cells, leading to mitochondrial fragmentation and apoptosis through the release of proapoptotic molecule cytochrome C (Choi *et al.*, 2005; Tiku *et al.*, 2020, 2021). OmpA further increases reactive oxygen species (ROS) levels and decreases mitochondrial polarization and ATP levels (Fig. 1). Concordantly *A. baumannii* OmpA mutant is significantly attenuated in its ability to induce host cell death (Choi *et al.*, 2005; Tiku *et al.*, 2021).

OmpA also helps in adherence and subsequent invasion of epithelial cells by interacting with extracellular matrix proteins such as fibronectin (Smani *et al.*, 2012). OmpA binds to factor H in human serum thereby assisting *A. baumannii* to escape complement-mediated killing (Kim *et al.*, 2009). Moreover, OmpA loss lowers the minimal inhibitory concentrations or MICs of multiple antibiotics, including chloramphenicol and aztreonam, which suggests that OmpA plays a key role in expelling antibiotics out of bacterial cells thereby leading to multidrug-resistant (MDR) phenotype of *A. baumannii* (Smani *et al.*, 2014). Another important porin protein linked with *A. baumannii* pathogenesis is the 33- to 36-kDa Omp called Omp33-36. This protein is also important for host cell adherence and its loss leads to attenuated host cell adhesion and lowered cytotoxicity in human lung epithelial cells and macrophages.

Omp33-36 loss reduces lethality, bacterial dissemination, and bacterial load in the lungs and spleen of mice (Smani *et al.*, 2013). Omp33-36 induces apoptosis by activating caspases and perturbs autophagy by causing an accumulation of the selective autophagy receptor p62/SQSTM1 and the autophagosomal membrane protein LC3B-II in immune and connective tissue cells. Modulation of autophagy enables *A. baumannii* to persist intracellularly and reside in autophagosomes (Rumbo *et al.*, 2014). Yet another important outer membrane porin protein Omp22 has been suggested to be an efficient target for vaccine development against *A. baumannii* infections. Immunization of mice with recombinant Omp22 increased survival rates of mice, reduced bacterial load in different organs, and lowered the levels of inflammatory cytokines and chemokines in the serum (Huang *et al.*, 2016).

The exact mechanism of how Omp22 functions in the pathogenesis of *A. baumannii* infections still remains elusive; nevertheless, Omp22 serving as a potential vaccine target holds promise in the race for developing therapeutics against *A. baumannii*. Additionally, OMVs, which are spherical vesicles secreted by the outer membrane of many Gram-negative bacteria, can also serve as a potent strategy by *A. baumannii* to deliver toxins. These toxins include OmpA, certain cytotoxic proteases and phospholipases (Kwon *et al.*, 2009; Tiku *et al.*, 2021).

Another important determinant of *A. baumannii* pathogenicity is the bacterial capsule. Mutants deficient in capsular exopolysaccharides are more susceptible to peptide antibiotics. Moreover, mutations that affect sugar precursors that lead to the formation of the capsule and lipopolysaccharide (LPS) sensitize *A. baumannii* to multiple antibiotics (Geisinger and Isberg, 2015). A transposon mutagenesis screen to identify genes required for proper growth of *A. baumannii* in inflammatory human ascites fluid revealed *ptk* and *epsA* as essential genes. Both *ptk* and *epsA* play important roles in capsule formation and assembly, and mutations in these genes lead to compromised capsule formation and growth defects of bacteria in human serum (Russo *et al.*, 2010). PglC is required for the formation of O-pentasaccharide found on the glycoproteins. Loss of *PglC* in *A. baumannii* prevented proper synthesis of the capsule, consequently leading to perturbed biofilm formation, and reduced virulence in mice (Lees-Miller *et al.*, 2013).

In addition to the components of the capsule, LPS, an immunoreactive molecule present in the outer membrane of Gram-negative bacteria, also leads to virulence of *A. baumannii. LpsB* glycotransferase is an essential factor required for the production of LPS in *A. baumannii,* and a mutation in this gene renders the bacterium defective in LPS, which causes decreased resistance to human serum and attenuated survival in rats (Luke *et al.*, 2010). A number of other studies have also highlighted the role of LPS in survival and pathogenicity of *A. baumannii* (Lin *et al.*, 2012; McQueary *et al.*, 2012).

Protein secretion systems form another potential virulent strategy used by *A. baumannii.* The bacterium possesses Type I, II, IV, V, and VI secretion systems, which have not been studied in great detail so far. Recently it was shown that *A. baumannii* uses its type 2 secretion system (T2SS) to secrete effector lipases LipA and LipH as well as the protease CpaA (Harding *et al.*, 2016). Another study from the same group reported the virulence potential of CpaA in both invertebrate and mouse model of pneumonia (Kinsella *et al.*, 2017). Furthermore *A. baumannii* utilizes T6SS for bacterial competition (Weber *et al.*, 2015), although its role in cytotoxicity is unknown yet.

## Host Responses Elicited by *A. baumannii*

**FIG. 2.**
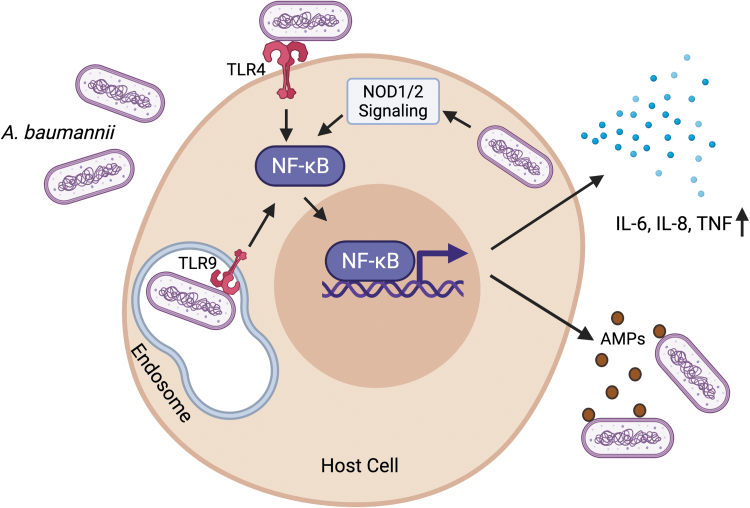
Host responses against *A. baumannii.* Infection with *A. baumannii* activates the plasma membrane-bound TLR4 and the endosome-bound TLR9. Activated TLR signaling leads to the activation and nuclear translocation of the transcription factor NF-κB. Intracellular *A. baumannii* also contributes to NF-κB activation through the intracellular PRRs—NOD1 and NOD2. Activated NF-κB drives the expression of proinflammatory cytokines, including TNF, and IL-6 and IL-8. NF-κB also stimulates the expression of AMPs like human β-defensins, which restrict *A. baumannii* growth. Figure created with BioRender.com. AMPs, antimicrobial peptides; IL, interleukin; PRRs, pathogen recognition receptors; TLR, toll-like receptor; TNF, tumor necrosis factor.

Multiple studies have uncovered specific host responses against *A. baumannii* infections. At the initial recognition of the bacteria, epithelial cells secrete antimicrobial peptides like human β-defensins that restrict *A. baumannii* growth, forming the first line of defense against the pathogen (Feng *et al.*, 2014). As with most infections, pattern recognition receptors (PRRs) at the cell membrane recognize pathogen-specific molecular signatures known as the pathogen-associated molecular patterns. A key PRR, membrane-bound toll-like receptor 4 (TLR4) has been implicated to play an important role in mounting an immune response and assisting bacterial clearance in *A. baumannii* infections in mice (Knapp *et al.*, 2006) (Fig. 2). Another PRR TLR2, which is important for recognizing peptidoglycan of the bacterial membrane, has a minimal role in host defense in curbing *A. baumannii* infections (Knapp *et al.*, 2006; Kim *et al.*, 2014).

Traditionally *A. baumannii* has been considered to be an extracellular pathogen, but recent evidence has shown that the bacterium can persist intracellularly as well (Sycz *et al.*, 2021). The pathogen invades epithelial cells and internalizes in a zipper-like mechanism associated with microtubule and microfilament-dependent uptake (Choi *et al.*, 2008). Internalized bacteria reside in membrane-bound vacuoles in the cytoplasm (Choi *et al.*, 2008), thereby imposing the need of detection by intracellular PRRs. Interestingly, TLR9, which is an endosomal PRR that detects viral/bacterial DNA and helps in mounting a subsequent immune response, is required for bacterial clearance and induction of proinflammatory cytokines and chemokines in a murine model of *A. baumannii* pneumonia (Noto *et al.*, 2015) (Fig. 2). Additionally, NOD1 and NOD2, which are also intracellular PRRs, and their serine/threonine adaptor protein kinase RIP2, which is induced upon *A. baumannii* infection, play central roles in sensing and subsequent clearance of *A. baumannii* in human airway epithelial cells (Fig. 2).

Loss of NOD1, NOD2, and RIP2 increases intracellular invasion and augments survival of *A. baumannii* in lung epithelial cells (Bist *et al.*, 2014). Finally sensing of the pathogen by PRRs leads to an activation of extensive downstream signaling, which involves concerted functions of NF-κB and the kinase cascade relayed by the mitogen-activated protein kinase pathway, ultimately culminating in the production of inflammatory molecules, including tumor necrosis factor, and interleukins (IL)-6 and IL-8 (March *et al.*, 2010; Bist *et al.*, 2014) (Fig. 2).

Cellular response of the host against *A. baumannii* entails recruitment of specialized immune cells to the site of infection. The migration of these cells is directed by cytokines and chemokines. Neutrophils and macrophages are the first cells to arrive (Harding *et al.*, 2017). Neutrophils are the major players in keeping *A. baumannii* infection under check by phagocytosing and clearing the bacteria from the infection site (Bhuiyan *et al.*, 2016). In accord with this, mice deficient in neutrophils exhibit acute lethality and a significant increase in bacterial burden upon *A. baumannii* infection (Faassen *et al.*, 2007; Breslow *et al.*, 2011). At the molecular level, neutrophils kill *A. baumannii* by using the NADPH phagocyte oxidase system, which leads to the production of reactive oxygen species (ROS). NADPH-deficient neutrophils are unable to contain bacterial replication and subsequent dissemination (Qiu *et al.*, 2009). Besides neutrophils, macrophages also help in the clearance of *A. baumannii* from the infection sites (Qiu *et al.*, 2012)*,* but the role of macrophages is secondary to neutrophils and is not vastly explored.

## Future Perspective

Extreme MDR phenotypes associated with *A. baumannii* have invoked great concern and have paved the way for studies aimed to understand the pathobiology of this bacterium. Future efforts should be focused on trying multiple strategies to combat *A. baumannii* infection. Clinical interventions like immunotherapy hold great promise for treatments against these infections. Since neutrophils are the major phagocytic killer cells in *A. baumannii* infections, methods of directing neutrophils toward the sites of infection using chemokines and cytokines maybe a viable therapeutic approach to consider. Additionally, other strategies like phage therapy, small molecules targeting bacterial metabolic pathways, and developing novel vaccine targets could prove beneficial (García-Quintanilla *et al.*, 2013). Since *A. baumannii* is resistant to most of the antibiotics currently available, clinicians opt for colistin as the last resort. However, recent cases have reported colistin resistance in *A. baumannii* as well. Therefore, it might be necessary to use combination therapies involving different antibiotics.

Finally, since *A. baumannii* possesses multiple protein secretion systems, it is of great interest to understand in detail their functions in regulating the virulence mechanisms of the pathogen. Many bacteria use their protein secretion systems to deliver potent virulence factors and toxins to spread infection. Therefore, it is vital to get a deeper understanding of the functioning of the different secretion systems in *A. baumannii* to develop successful therapeutics against this pathogen.
